# Massive Hypopharyngeal Dilatation and Cervical Lung Herniation in a Semi-Professional Wind Instrument Player: Highlighting the Necessity of Personalized Diagnostic and Management Strategies

**DOI:** 10.3390/jpm16030127

**Published:** 2026-02-25

**Authors:** Michail Galanis, Florian Dammann, Konstantinos Gioutsos, Patrick Dorn, Eberhard Seifert

**Affiliations:** 1 Department of Thoracic Surgery, Inselspital, University Hospital of Bern, 3010 Bern, Switzerland; konstantinos.gioutsos@insel.ch (K.G.); patrick.dorn@insel.ch (P.D.); 2 Department of Diagnostic, Interventional and Pediatric Radiology, Inselspital, Bern University Hospital and University of Bern, 3010 Bern, Switzerland; florian.dammann@insel.ch; 3 Division of Phoniatrics, ENT Clinic, Head and Neck Surgery, University of Bern, Inselspital, 3010 Bern, Switzerland; eberhard.seifert@insel.ch

**Keywords:** pharyngocele, laryngocele, personalized medicine, precision medicine, lung herniation, hypopharyngeal dilatation, wind instrument player, occupational medicine, dynamic imaging, individualized treatment

## Abstract

Wind instrument performance requires sustained and repetitive increases in intrathoracic and pharyngeal pressures, which may lead to rare but clinically relevant anatomical alterations of the upper aerodigestive tract. We report the case of a 46-year-old male semi-professional wind instrument player who developed massive hypopharyngeal dilatation and cervical lung herniation as a consequence of long-term, high-pressure musical activity. Dynamic imaging performed during instrument playing demonstrated marked hypopharyngeal expansion and herniation of the lung apices into the cervical region, highlighting the importance of individualized diagnostic strategies that replicate patient-specific triggers. Multidisciplinary evaluation integrating otorhinolaryngology, thoracic surgery, radiology, and pulmonology led to a personalized risk assessment and the recommendation to cease wind instrument performance in order to prevent potentially life-threatening complications, such as pneumothorax. This case illustrates how personalized diagnostic approaches and tailored clinical decision-making are essential in managing rare occupational conditions. A comprehensive review of the literature is provided, with a focus on individualized risk factors, diagnostic strategies, and personalized treatment concepts relevant to precision medicine.

## 1. Introduction

Wind instrument performance requires complex coordination of respiratory mechanics, glottic control, and upper aerodigestive tract function. Depending on the instrument and playing technique, substantial and repetitive increases in intrathoracic and intrapharyngeal pressure may be generated, particularly during sustained high-intensity performance and prolonged practice sessions [1,2,3]. While the majority of musicians tolerate these physiological demands without complications, occupational exposure over years may contribute to pressure-associated disorders of the upper aerodigestive tract in susceptible individuals [1,4,5].

Pharyngoceles and laryngoceles are rare structural abnormalities that may be associated with increased intraluminal pressure and chronic mechanical strain [6,7,8]. Pharyngoceles are classically described as mucosal herniations of the pharyngeal wall, often through the thyrohyoid membrane, and are frequently reported in association with repeated Valsalva-like maneuvers, including wind instrument playing and glassblowing [6,7,9,10]. Laryngoceles represent dilatations of the laryngeal saccule and may present as internal, external, or mixed lesions, with clinical manifestations ranging from incidental findings to neck swelling, hoarseness, recurrent infections, or airway compromise [8,11,12,13,14,15]. Although these entities are benign, they may cause significant functional impairment and in rare cases lead to complications requiring urgent intervention [12,14].

The diagnosis of pressure-related pharyngolaryngeal disorders may be challenging because symptoms and anatomical changes can be intermittent and occur predominantly under dynamic conditions. Standard static imaging or resting endoscopic examinations may therefore underestimate the extent or functional relevance of disease. Dynamic diagnostic approaches, including endoscopy and cross-sectional imaging performed during Valsalva-like maneuvers or symptom-provoking activity, can provide essential additional information in such cases [3,9,10].

Despite increasing awareness, the evidence base for both pharyngoceles and laryngoceles remains limited and is largely composed of case reports and small case series, which restricts robust comparisons of therapeutic outcomes and standardized clinical decision-making [6,8,9,10,15]. Nevertheless, reported management strategies range from conservative measures such as behavioral modification and avoidance of pressure-provoking triggers to surgical interventions in symptomatic, complicated, or progressive cases [13,16,17,18,19].

Here, we report a rare combined constellation in a semi-professional wind instrument player, characterized by massive hypopharyngeal dilatation associated with cervical lung herniation, documented under dynamic symptom-provoking conditions. This case highlights the importance of individualized diagnostic strategies and personalized risk assessment in occupational pressure-related disorders and underscores the potential relevance of dynamic imaging for identifying clinically meaningful anatomical relationships and complication risks [3,7,9,10,15].

## 2. Case Presentation

The patient was a 46-year-old male semi-professional wind instrument player who presented with acute, sharp, right-sided laryngeal pain following an intensive session of bagpipe playing. He had a more than 25-year history of regular wind instrument performance, including the bagpipe, euphonium, and clarinet. His musical activity involved prolonged daily practice sessions requiring sustained and repetitive generation of high intrathoracic and intrapharyngeal pressures, representing a highly individualized occupational exposure profile.

The patient’s medical history was notable for a prior burnout syndrome ten years earlier, which was treated with escitalopram. At presentation, he was taking escitalopram 10 mg daily, diclofenac as needed for pain, and pantoprazole, which had recently been increased to 40 mg twice daily due to persistent laryngopharyngeal reflux (LPR) symptoms. He reported no drug allergies, was a lifelong non-smoker, consumed little alcohol, and had no relevant family history. Overall, the patient exhibited a low baseline pulmonary risk profile, emphasizing the occupational nature of his condition.

His primary complaint was a one-month history of reproducible, sharp, right-sided laryngeal pain that occurred exclusively during wind instrument playing and typically developed several minutes after initiating performance. He denied dysphonia, dyspnea, dysphagia, or exercise intolerance. In contrast, he reported daily symptoms of LPR, particularly postprandially, which was considered a relevant patient-specific cofactor contributing to mucosal vulnerability.

### 2.1. Physical Examination

Upon physical examination, the patient appeared well, with normal vital signs and no immediate distress. An ENT examination revealed a slight facial asymmetry, with the right mandible appearing slightly larger than the left. There were no visible neck masses or swelling observed at rest. Examination of the oropharynx showed normal mucosal surfaces without any visible lesions. Laryngeal palpation revealed no tenderness or palpable masses. The tonsils showed no pathological findings. The absence of findings at rest highlighted the dynamic and activity-dependent nature of the patient’s symptoms.

### 2.2. Diagnostics

#### 2.2.1. Fiberoptic Endoscopy/Laryngostroboscopy

Laryngostroboscopy was performed, which demonstrated that the larynx was morphologically normal with symmetrical vocal cord movement. However, there was a sign of LPR observed on the posterior pharyngeal wall. Edema was noted in the interarytenoid region and at the esophageal sphincter. There was also slight asymmetry observed in the right piriform sinus, which appeared more voluminous than the left side, potentially correlating with the patient’s symptoms during instrument playing.

To achieve a personalized diagnostic assessment, fiberoptic endoscopy was performed during active wind instrument playing using different instruments with varying pressure requirements, including the pan flute, euphonium, and bagpipe. The results showed a progressive increase in the size of the dorsal pharyngocele as the instrument’s required intrathoracic pressure increased. The pharyngocele was smallest when playing the pan flute and largest when playing the bagpipe, indicating a direct correlation between the pressure requirements of each instrument and the degree of pharyngeal dilatation (Figure S1).

#### 2.2.2. Dynamic CT-Scan-Imaging

To further investigate the patient’s symptoms, dynamic CT imaging was performed both at rest and during wind instrument playing to assess changes in anatomical structures. The scans taken at rest demonstrated a relatively large hypopharyngeal volume with no abnormal masses or lesions. However, during bagpipe playing, the imaging revealed significant anatomical alterations. There was massive dilatation of the hypopharynx, most notably on the right side. Bilateral herniation of the thyrohyoid membrane into the neck’s soft tissues was observed, along with significant dilatation of the posterior and lateral walls of the hypopharynx. The right-sided mucosal fold protruded into the dilated hypopharyngeal lumen (Figures S2 and S3).

Additionally, CT imaging revealed cranial displacement of both lung apices into the cervical region, more pronounced on the right side. The right lung apex was displaced ventrally, resulting in a 12 mm deviation of the esophagus up to the level of the first thoracic vertebra (Th-1). A thin separating membrane of approximately 2.5 mm between the right piriform recess and the lung apex indicated a critically reduced anatomical barrier, representing a patient-specific risk factor for pneumothorax.

#### 2.2.3. Multidisciplinary Consultation

Given the rarity and complexity of the findings, a multidisciplinary team including otorhinolaryngologists, thoracic surgeons, radiologists, and pulmonologists conducted an individualized risk assessment. The team concluded that the patient’s condition—massive hypopharyngeal dilatation and cervical lung herniation—was primarily caused by the sustained high intrathoracic pressures associated with wind instrument playing.

Despite preserved baseline pulmonary function, the markedly reduced tissue separation between the hypopharynx and lung apex was considered to confer a high individualized risk for overpressure-related complications, particularly pneumothorax.

### 2.3. Diagnosis and Personalized Management

The patient was diagnosed with a massive dilatation of the posterior and lateral walls of the hypopharynx. Additionally, he was diagnosed with cervical lung herniation of the lung apices, more pronounced on the right side. LPR was identified as a contributing factor, exacerbating mucosal irritation in the pharyngeal region.

Therapeutic management was guided by an individualized risk assessment integrating anatomical findings, occupational exposure, and potential complication risk. Given the markedly reduced anatomical separation between the hypopharynx and the lung apex, continued exposure to high intrathoracic pressure during wind instrument playing was considered to confer a high personalized risk for overpressure-related complications, including pneumothorax. Consequently, immediate cessation of wind instrument performance was recommended as the primary preventive measure.

Conservative medical management was tailored to address modifiable patient-specific factors. Antireflux therapy was intensified with pantoprazole 40 mg twice daily to reduce ongoing mucosal inflammation and minimize additional mechanical vulnerability of the hypopharyngeal tissues. In parallel, the patient was referred for a personalized physiotherapy program consisting of nine sessions, focusing on the reduction of cervical and prelaryngeal muscle tension, with the aim of alleviating secondary muscular strain and optimizing regional biomechanics.

Structured clinical follow-up was initiated to allow individualized monitoring for symptom progression, particularly the development of respiratory compromise or swallowing difficulties. The patient received detailed education regarding early warning signs of acute complications, including sudden dyspnea or chest pain, and was instructed to seek immediate medical attention should such symptoms occur.

Given the profound professional and emotional impact of the recommendation to discontinue wind instrument playing, patient-centered counseling and shared decision-making were integral components of care. Alternative activities and coping strategies were discussed in order to preserve quality of life while prioritizing patient safety. This personalized management approach reflects the necessity of integrating anatomical, functional, and psychosocial factors when treating rare occupational disorders.

### 2.4. Management Rationale

Given the pressure-dependent and occupational nature of the findings, management was discussed in a multidisciplinary setting and guided by individualized risk assessment and shared decision-making. As the patient had preserved pulmonary function, no fixed airway obstruction at rest, no recurrent infection, and no acute complication (e.g., pneumothorax or progressive respiratory compromise), an initial conservative approach was considered appropriate [3,8,15]. In addition, because ongoing high-pressure exposure during wind instrument playing was identified as the principal modifiable trigger, behavioral modification with reduction/avoidance of symptom-provoking playing technique was prioritized as a first-line strategy [3,9,10]. Surgical options were discussed with the patient as a potential escalation strategy in the event of persistent symptoms, progression, recurrent infection, functional impairment, or complication risk, consistent with the predominantly case-based evidence available in the current literature [13,15,16,19].

## 3. Discussion

Pharyngoceles and laryngoceles are uncommon structural abnormalities affecting the pharynx and larynx, respectively, and are generally considered pressure-associated disorders of the upper aerodigestive tract. They are often related to repeated increases in intrapharyngeal and intrathoracic pressures, which may occur during activities such as playing wind instruments (particularly brass instruments), glassblowing, or other forms of sustained forced expiration [1,2,3,4,5].

Pharyngoceles are typically described as outpouchings of pharyngeal mucosa through the thyrohyoid membrane. Clinical presentation can range from intermittent neck swelling, dysphagia, cough, and discomfort to mild respiratory symptoms. However, many cases remain asymptomatic and are discovered incidentally, potentially delaying diagnosis [6,7,9,10]. Pharyngoceles have been increasingly discussed as an occupational overuse syndrome in individuals exposed to repetitive high-pressure maneuvers, and modern imaging plays a key role in confirming the diagnosis [9,10].

Laryngoceles represent dilatations of the laryngeal saccule and may be classified as internal, external, or mixed lesions. They may remain asymptomatic, but symptomatic patients can present with neck swelling, hoarseness, cough, dyspnea, or airway compromise. Complications such as infection can cause acute deterioration and even airway obstruction, highlighting the potential severity despite their benign nature [8,12,14]. Laryngoceles may also occur in the absence of classic risk factors, suggesting that underlying anatomical predisposition may contribute in selected patients [11].

The published literature differs in density and scope. Pharyngoceles are exceptionally uncommon and are mostly reported in isolated case reports or small series, limiting standardized management recommendations [6,9,10]. Laryngoceles are described more frequently and have been reviewed in larger overviews, yet evidence remains predominantly based on case reports and retrospective summaries [8,13,15]. This restricts direct comparison of surgical outcomes and complicates evidence-based recommendations [15].

The present case expands the spectrum by demonstrating a rare combined constellation rather than an isolated pathology. The combination of massive hypopharyngeal dilatation and cervical lung herniation appears highly unusual in a semi-professional wind instrument player. Most published cases describe either isolated pharyngoceles or isolated laryngoceles, while complex multi-compartment pathology remains sparsely reported [6,8,9,10,11]. Importantly, lesions were documented under dynamic symptom-provoking conditions, which is clinically relevant given the intermittent and pressure-dependent nature of these entities [3,9,10].

Dynamic imaging has key diagnostic advantages in occupational pressure-related disorders. Static imaging may underestimate disease severity or fail to capture transient anatomical changes, whereas imaging during Valsalva-like maneuvers or simulated activity may demonstrate the true extent of herniation and functional implications [3,9,10]. This aligns with publications emphasizing imaging-based confirmation and occupational mechanisms [9,10].

A unique and clinically concerning aspect of this case was the critically reduced anatomical barrier of approximately 2.5 mm between hypopharyngeal structures and adjacent pulmonary tissue. This proximity supports a patient-specific risk-based interpretation, as minimal separation may increase vulnerability to pulmonary complications under extreme mechanical stress [4]. Serious complications of pressure-related pharyngolaryngeal disorders may include recurrent infections, aspiration, and rare potentially life-threatening events depending on anatomy and stress distribution [4].

Management of pharyngoceles and laryngoceles is symptom-driven and individualized. Conservative treatment is generally appropriate for asymptomatic or mildly symptomatic patients and typically includes behavioral modifications and management of contributing factors [3,9,18]. Surgical intervention may be indicated in cases with functional impairment, progressive disease, recurrence, infection, airway obstruction, or high complication risk [12,14,15,16,19].

For laryngoceles, both external and endoscopic approaches have been described, including modern endoscopic and evolving techniques depending on lesion type and institutional expertise [13,15,17]. Recent literature also discusses management strategies in the context of contemporary surgical approaches, although evidence remains non-comparative [15]. Several reports describe successful operative treatment in complex settings including infection or mixed lesions, but systematic comparison remains limited [12,14,20].

In summary, pharyngoceles and laryngoceles are rare but clinically relevant pressure-associated disorders of the upper aerodigestive tract. They occur most typically in individuals exposed to repetitive high pressure, but may also arise without classical triggers, suggesting anatomical predisposition [1,4,5,11]. Early recognition, risk stratification, and appropriate use of dynamic diagnostic tools are particularly important when occupational exposure is involved and when anatomical proximity raises concern for pulmonary complications [3,4,9,10].

### 3.1. Implications for Personalized and Precision Medicine

This case illustrates how rare pressure-related disorders of the upper aerodigestive tract require an individualized, precision medicine–oriented approach for accurate diagnosis and safe management. In our patient, the severity and functional relevance of hypopharyngeal dilatation and cervical lung herniation could not have been sufficiently characterized using standard static examinations alone. Patient-specific diagnostic strategies, including dynamic endoscopy and computed tomography performed during symptom-provoking wind instrument activity, were essential to demonstrate the true pathology and guide individualized clinical risk assessment [3,9,10].

From a personalized medicine perspective, several individual factors influenced risk stratification and clinical decision-making, including long-term occupational exposure to sustained intrathoracic pressure, instrument-specific pressure demands, subtle anatomical vulnerability, and concomitant LPR. These factors collectively created a risk profile that differs substantially from asymptomatic wind instrument players or patients with incidental isolated pharyngoceles or laryngoceles [1,2,3,9].

Therapeutic management was likewise individualized. Although surgical treatment is discussed in the literature and may be appropriate in selected symptomatic or complicated cases, conservative management was chosen in this patient based on preserved pulmonary function, absence of fixed airway obstruction, and identification of continued high-pressure exposure as the principal modifiable trigger [3,8,15]. In addition, shared decision-making was particularly important given the professional and emotional impact of recommending reduction or cessation of wind instrument playing.

Overall, this case supports the relevance of precision medicine frameworks in evaluating rare occupational disorders. Future research should aim to define susceptibility markers, establish standardized dynamic diagnostic protocols, and develop personalized risk models to guide prevention and tailored therapeutic strategies for high-risk populations [9,15].

### 3.2. Follow-Up Considerations, Risk Stratification, and Clinical Monitoring

Beyond confirming the diagnosis, this case also highlights how important an individualized follow-up strategy can be in rare, pressure-dependent conditions of the upper aerodigestive tract. A key difficulty is that many of these patients may appear clinically stable during routine assessment, because symptoms only occur under very specific circumstances. In our patient, there were no relevant complaints at rest, and no constant airway compromise. However, dynamic imaging clearly demonstrated pronounced anatomical changes once the triggering activity was reproduced. This underlines that “doing well” in everyday life does not necessarily mean that the overall risk is negligible, particularly when occupational exposure repeatedly provokes extreme physiological strain.

In this case, one of the most striking features was the very small tissue separation between the hypopharyngeal structures and the cranially displaced lung apex. Although pneumothorax is not a widely described direct complication of hypopharyngeal dilatation, the close anatomical proximity raises a plausible concern that pressure-related events could theoretically become more likely, especially during sustained high-pressure expiration. The fact that symptoms were consistently reproducible and strictly linked to wind instrument performance further supports a cautious approach. Even without previous acute complications, this combination of extreme dynamic change and individual anatomy may represent a patient-specific vulnerability marker, and should be considered when discussing preventive recommendations.

Another practical observation from this case is that the extent of the dynamic deformation seems to vary substantially depending on the instrument played and the technique required. The clear pressure-related gradient we observed during endoscopy with different instruments suggests that the risk may not be uniform across wind instrument players. In clinical routine, this may open a pragmatic path toward harm reduction rather than absolute restrictions in all cases. For example, some patients may benefit from limiting high-pressure instruments, shortening practice sessions, planning structured breaks, or adapting technique. However, in situations like ours, where deformation is pronounced and the anatomical “buffer zone” is minimal, avoiding the highest-pressure exposure may still be the safest recommendation. Importantly, these decisions should be made in a shared decision-making process, acknowledging the uncertainties and the lack of clear evidence-based thresholds in such rare presentations.

We also considered the potential contribution of concomitant LPR. Chronic reflux-related inflammation may reduce mucosal resilience and could, at least in theory, promote susceptibility to pressure-driven distension over time. While this association remains speculative, reflux represents a modifiable factor and can be addressed with comparatively low risk. Optimizing reflux management may therefore not only improve local symptoms but also reduce ongoing mucosal irritation that might otherwise contribute to progression. In rare disorders where prospective data are unavailable, treating plausible and treatable cofactors is often a reasonable part of an individualized prevention strategy.

A further challenge is defining what “progression” actually means in a clinically meaningful way. Traditional endpoints such as fixed airway compromise at rest may only appear late. For that reason, follow-up should not be based solely on resting symptoms but should instead focus on changes in trigger-related complaints and functional limitations. Repeat evaluation may be appropriate if symptoms worsen, if new dysphagia or aspiration signs emerge, if pain becomes more frequent, or if activity modification fails to reduce provocation. While repeated CT imaging should be balanced against radiation exposure, repeat dynamic assessment can be valuable when clinical changes occur. Depending on the situation, follow-up might include endoscopic reassessment under controlled provocation, pulmonary evaluation if respiratory symptoms develop, or targeted imaging after multidisciplinary discussion. This case reinforces the benefit of close collaboration between ENT, radiology, pulmonology, and thoracic surgery perspectives when symptoms and anatomy overlap in an unusual way.

Finally, the human side of these recommendations should not be underestimated. For many musicians, wind instrument performance is not simply a leisure activity, but a major part of personal identity, social life, and in some cases professional stability. Advising someone to reduce or stop playing can therefore carry a significant emotional and occupational burden. In our view, individualized management should explicitly address this aspect, ensuring that counseling remains empathetic, realistic, and supportive. Where possible, alternative approaches such as adjusting repertoire, exploring lower-pressure instruments, or adapting performance habits may help preserve quality of life while prioritizing safety. Ultimately, this case illustrates that individualized medicine is not only about anatomy and imaging, but also about aligning clinical recommendations with patient priorities in a transparent and responsible way.

## 4. Conclusions

This case demonstrates that sustained, patient-specific exposure to elevated intrathoracic and intrapharyngeal pressures during wind instrument playing can result in rare but clinically significant anatomical alterations, including massive hypopharyngeal dilatation and cervical lung herniation. Such conditions may remain undetected by routine examinations performed at rest and require individualized diagnostic strategies for accurate assessment.

A personalized medicine approach was essential in this case, combining dynamic, activity-adapted imaging with interdisciplinary evaluation to define an individualized risk profile. Despite preserved baseline pulmonary function, patient-specific anatomical features, particularly the minimal separation between the hypopharynx and lung apex, indicated a high risk for pressure-related complications, including pneumothorax.

Management decisions were therefore guided by personalized risk–benefit assessment rather than standardized treatment algorithms. Tailored conservative management, combined with patient-centered counseling and shared decision-making, allowed for the prevention of potentially life-threatening complications while addressing the patient’s occupational and psychosocial context.

This report underscores the importance of precision medicine in the evaluation and management of rare occupational disorders and highlights the need for individualized diagnostic and therapeutic strategies. Future studies should focus on identifying patient-specific susceptibility factors and developing personalized risk assessment models for individuals exposed to chronic intrathoracic pressure.

## Data Availability

The raw data supporting the conclusions of this article will be made available by the authors on request.

## Supplementary Materials

The following supporting information can be downloaded at: https://www.mdpi.com/article/10.3390/jpm16030127/s1: Figure S1: Laryngostroboscopy findings, Figure S2: Dynamic CT-scan reveals a massive pharyngocele and a lung herniation to the neck, Figure S3: Massive dilatation of hypopharynx and herniation of the Membrana thyrohyoidea.

## Abbreviations

The following abbreviations are used in this manuscript:

ENT	Ear–Nose–Throat
LPR	Laryngopharyngeal reflux
CT	Computer Tomography
Mm	Millimeter
Th	Thoracic
