# Supplementary material for: Massive Hypopharyngeal Dilatation and Cervical Lung Herniation in a Semi-Professional Wind Instrument Player: Highlighting the Necessity of Personalized Diagnostic and Management Strategies
Source: J Pers Med. 2026 Feb 25;16(3):127. doi: 10.3390/jpm16030127 (PMC13028340; doi:10.3390/jpm16030127)
Supplement: Supplementary file 1 [file jpm-16-00127-s001.zip › jpm-4078838-supplementary.pdf]

Supplementary files

Figures:

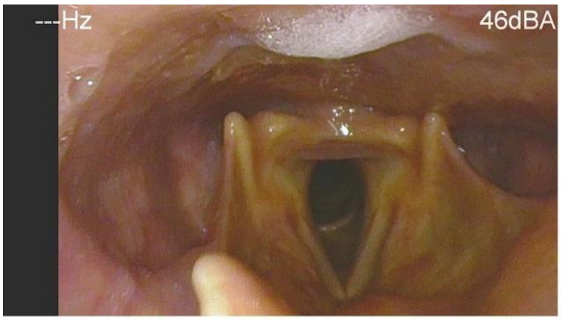

Resting breathing

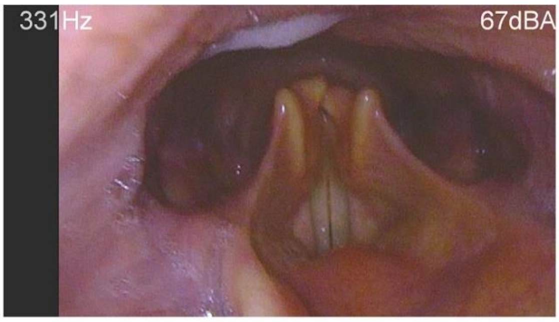

During phonation, the piriform sinuses on both sides are visible; the right piriform sinus appears slightly more voluminous.

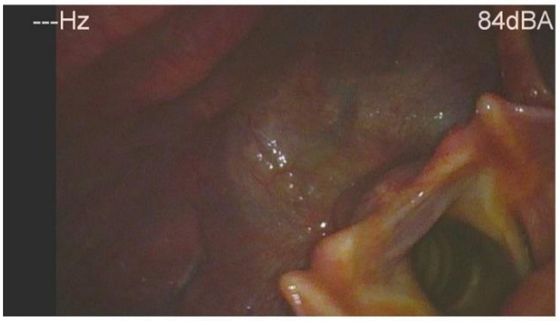

During bagpipe playing, a balloon-like inflated laryngo-pharyngocele is observed dorsal to the larynx.

Figure 1: Laryngostroboscopy findings.

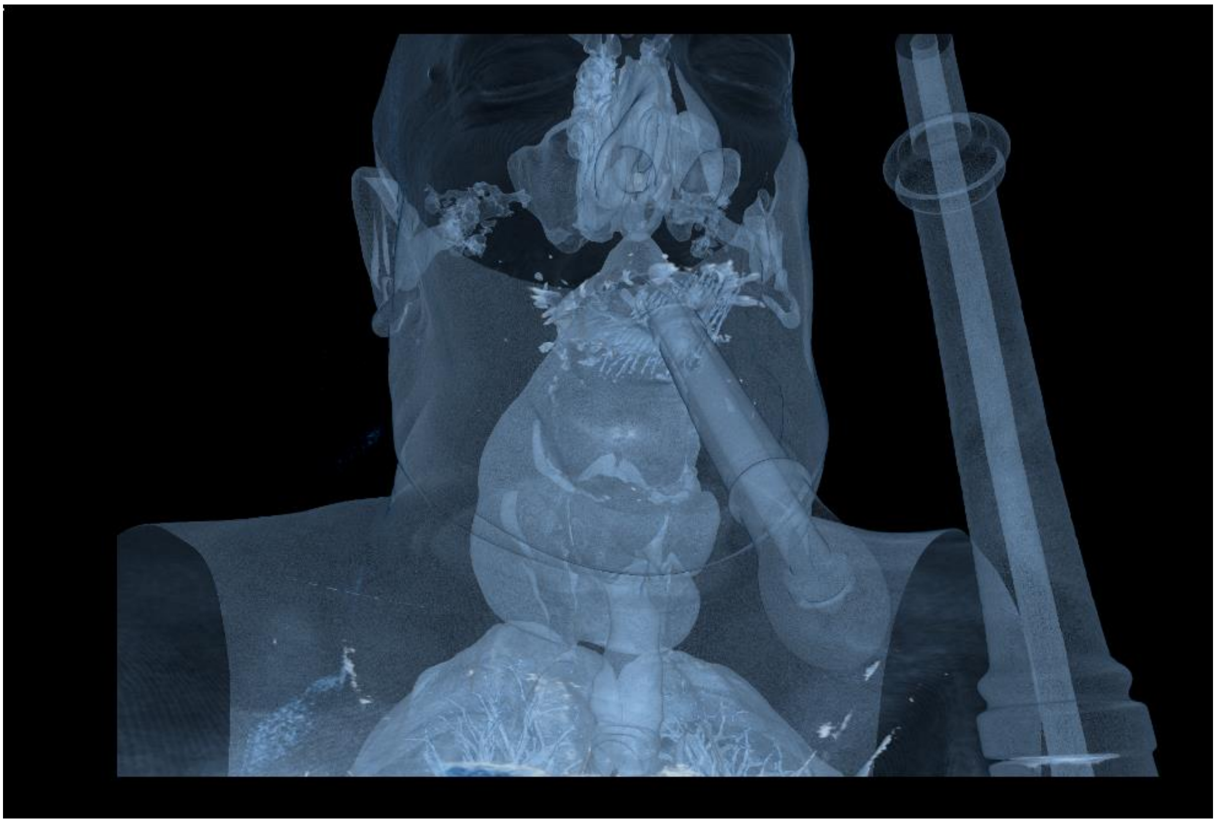

Figure 2: Dynamic CT-scan reveals a massive pharyngocele and a lung herniation to the neck.

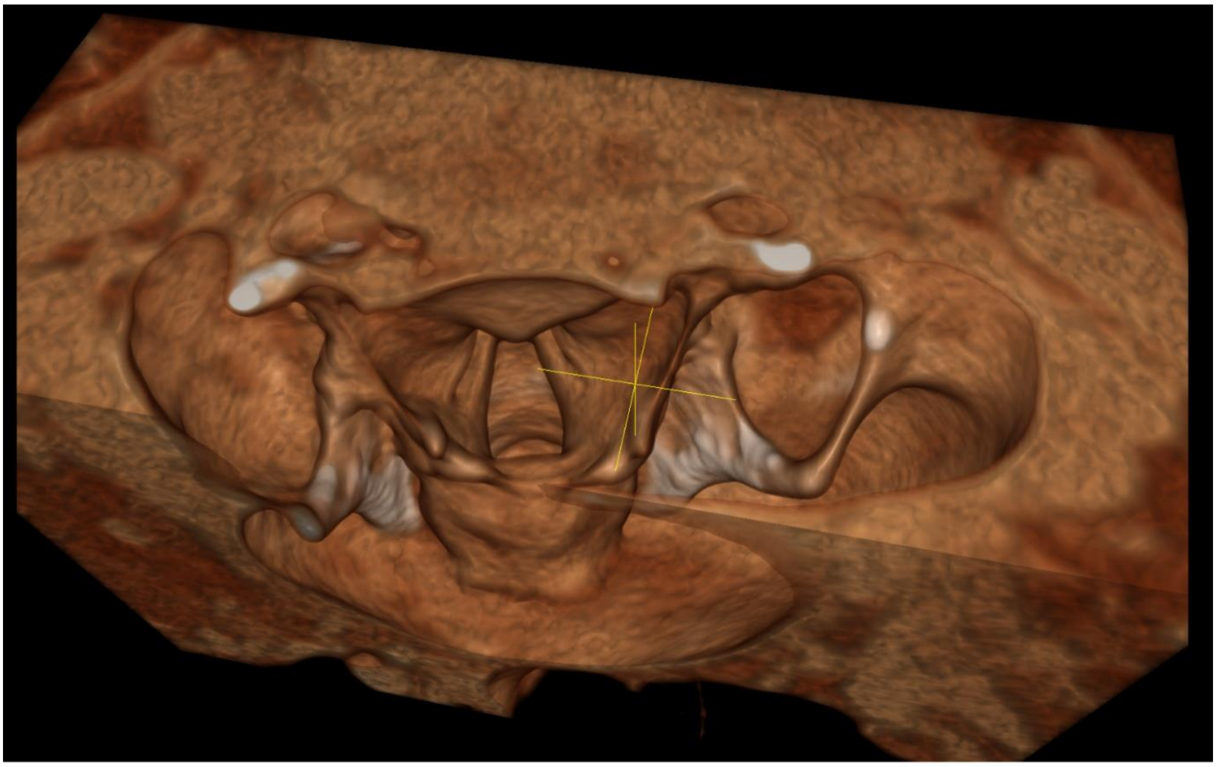

Massive Dilatation des Hypopharynx mit Hernierung der Membrana thyrohyoidea rechts mehr als links.

Figure 3: Massive dilatation of hypopharynx and herniation of the Membrana thyrohyoidea.
